# Full length and protease domain activity of chikungunya virus nsP2 differ from other alphavirus nsP2 proteases in recognition of small peptide substrates

**DOI:** 10.1042/BSR20150086

**Published:** 2015-05-27

**Authors:** Chonticha Saisawang, Pornpan Sillapee, Kwanhathai Sinsirimongkol, Sukathida Ubol, Duncan R. Smith, Albert J. Ketterman

**Affiliations:** *Institute of Molecular Biosciences, Mahidol University, Salaya Campus, Thailand; †Center for Emerging and Neglected Infectious Diseases, Mahidol University, Thailand; ‡Department of Microbiology, Faculty of Science, Mahidol University, Bangkok, Thailand

**Keywords:** alphavirus, chikungunya virus, cysteine protease, non-structural protein 2 (nsP2), CHIKV, chikungunya virus, Dnp, 2,4-dinitrophenyl, MBP, maltose binding protein, Nma, 2-(*N*-methylamino)benzoyl, cleavage sites nsP1/nsP2, AGA, nsP2/nsP3, AGC, nsP3/nsP4, AGG, nsP2, non-structural protein 2, nsP2-FL/Pro, full length/protease domain only, SFV, Semliki forest virus, SINV, Sindbis virus, VEEV, Venezuelan equine encephalitis virus

## Abstract

Alphavirus nsP2 proteins are multifunctional and essential for viral replication. The protease role of nsP2 is critical for virus replication as only the virus protease activity is used for processing of the viral non-structural polypeptide. Chikungunya virus is an emerging disease problem that is becoming a world-wide health issue. We have generated purified recombinant chikungunya virus nsP2 proteins, both full length and a truncated protease domain from the C-terminus of the nsP2 protein. Enzyme characterization shows that the protease domain alone has different properties compared with the full length nsP2 protease. We also show chikungunya nsP2 protease possesses different substrate specificity to the canonical alphavirus nsP2 polyprotein cleavage specificity. Moreover, the chikungunya nsP2 also appears to differ from other alphavirus nsP2 in its distinctive ability to recognize small peptide substrates.

## Abstract 2

The protease role of alphavirus nsP2 is critical for virus replication as only the virus protease processes the viral non-structural polypeptide. We show chikungunya nsP2 protease possesses different substrate specificity to the canonical alphavirus nsP2 polyprotein cleavage specificity.

## INTRODUCTION

Chikungunya virus (CHIKV), the causative agent of chikungunya fever, is an *Alphavirus* belonging to the family *Togaviridae*, that was originally isolated in Tanzania, Africa [1]. In Africa, CHIKV is believed to be maintained in a sylvatic transmission cycle between forest dwelling mosquitoes and wild non-human primates such as vervet monkeys or baboons [2,3]. In Asia and elsewhere transmission is generally maintained in an urban human–mosquito–human cycle [4] with *Aedes* genus mosquitoes being the major vectors, especially *Ae. aegypti* and *Ae. albopictus*. Although *Ae. albopictus* or the Asian tiger mosquito is a native species of Southeast Asia, it was involved in a large chikungunya outbreak in the Indian Ocean islands in 2005–2006 [5]. A mutation of the CHIKV E1 structural protein (Ala226Val) was observed at the beginning of the outbreak and it was subsequently found that the E1 mutation enhanced infectivity in *Ae. albopictus* mosquitoes [6]. Regrettably, *Ae. albopictus* is one of the world's 100 most invasive species, increasing the risk of CHIKV emerging or re-emerging and becoming a major health problem around the world [4]. This has been exemplified by the recent introduction of CHIKV into the Americas, where, within a short time, more than a million suspected cases of chikungunya fever are believed to have occurred [7]. Chikungunya fever is typically characterized by headache, high fever, skin rash and nausea as well as joint pain that can be severe and long lasting, and, although rare, fatalities have been reported [8].

CHIKV is comprised of a genome of an approximately 12 kb positive sense single-stranded RNA including a 5′cap and 3′poly(A) tail [9]. Almost two-thirds of the RNA genome encodes for the non-structural polyprotein precursor, nsP1234, which is processed by the virally encoded protease (nsP2) activity generating the replication complex. Initial processing generates nsP4 and nsP123 which direct synthesis of the negative sense template RNAs, while subsequent processing into nsP1 and nsP23 generates a complex that produces both sense and anti-sense RNAs. Final processing generates discrete nsP2 and nsP3 proteins [10]. The replication complex additionally transcribes a small 26S subgenomic RNA from the 3’-end of the genome that encodes the three structural proteins (Capsid, E1 and E2) as well as two small accessory proteins which are subsequently processed by viral and host cell proteases [4].

In addition to its protease activity, the viral nsP2 protein possesses multiple enzymatic functions including RNA helicase, nucleoside triphosphatase (NTPase) and RNA-dependent 5′-triphosphatase activities [11–13]. The protease domain is located at the C-terminus of the nsP2 protein, and it is postulated that the CHIKV nsP2 is a papain-like cysteine protease although there is no direct experimental evidence to support this statement [11,14]. However, other alphaviruses such as Sindbis virus (SINV), Semliki forest virus (SFV) and Venezuelan equine encephalitis virus (VEEV) have been well-studied, and the nsP2 protein has been experimentally characterized as a cysteine protease [14–18]. Primary amino acid alignment of the CHIKV nsP2 protease with the other alphaviruses shows the presence of the conserved cysteine and histidine catalytic dyad [18,19], and the protease active site residues are highly conserved across the different alphaviruses.

Presently, only a truncated CHIKV nsP2, consisting of the isolated protease domain (nsP2-Pro) has been proteolytically characterized [11]. In this study we have biochemically characterized the protease activity of the full-length nsP2 protein and compared it to the activity of the isolated protease domain.

## METHODOLOGY

### DNA construction

Chikungunya viral RNA was prepared from virus stocks of a previously described Thai CHIKV (ECSA E1:226V) isolate [20] using the illustra RNAspin mini RNA isolation kit (GE Healthcare). First-strand cDNA was synthesized by reverse transcription and was then used as a template for PCR amplification. Specific primers were used to amplify the 2394 nucleotides of the full length nsP2 protein (nsP2-FL) and the 1131 nucleotides of the protease domain of nsP2 (nsP2-Pro). Both sequences were cloned into an engineered vector derived from the pET21d vector. This vector was engineered to contain the maltose binding protein (MBP), a tobacco etch virus (TEV) protease recognition site, an 8xHis-tag and the PreScission protease recognition site, respectively. Moreover this vector was also designed to have a SmaI restriction site to facilitate the cloning by the Ligation Independent Cloning (LIC) method. The recombinants were transformed into *Escherichia coli* DH5α, and the candidate clones were verified by DNA sequencing.

### Protein expression and purification of the nsP2 proteins

The nsP2 recombinant plasmids were extracted and re-transformed into *E. coli* BL21-CodonPlus® (DE3)-RIL competent cells. The cells were cultured in terrific broth supplemented with 100 μg/ml ampicillin and 34 μg/ml chloramphenicol at 37°C until the cell density reached 0.5 OD_600nm_. Protein expression was induced with 0.2 mM isopropyl-β-D-thiogalactopyranoside (IPTG) at 18°C in overnight culture. The cells were subsequently centrifuged and kept at -20°C until use. The nsP2-FL and nsP2-Pro proteins were purified by the same protocol. The cell pellets were resuspended with 50 mM Tris/HCl, pH 7.5, 200 mM NaCl, 0.2 M arginine and 0.2 M glutamic acid (equilibration buffer). The cells were chemically lysed by the addition of 2 mg/ml lysozyme and physically lysed by sonication and centrifuged at 10,000 ***g*** for 30 min at 4°C. The supernatants were filtered through 0.45 μm membranes before being applied to HisTrap HP columns (GE Healthcare). After application, the columns were washed with 20 column volumes of equilibration buffer followed by 10 column volumes of 20 mM imidazole in equilibration buffer. The MBP-His tag was cleaved with PreScission protease by on-column cleavage at room temperature for 30 min. The cleaved proteins were eluted from the columns and applied to 5 ml HiTrap desalting columns (GE Healthcare) for buffer exchange to a cation exchanger buffer (50 mM potassium phosphate, pH 6.5, 0.2 M arginine and 0.2 M glutamic acid). The cation exchanger columns (1 ml HiTrap™ SP-XL) were used to remove the remaining contaminants. The bound proteins were eluted with 50 mM potassium phosphate, pH 6.5, 0.2 M arginine, 0.2 M glutamic acid and 200 mM NaCl. Finally, the pure proteins were desalted and kept in 50 mM Tris/HCl, pH 7.5, containing 0.2 M arginine and 0.2 M glutamic acid.

Protein concentrations were determined using Bradford reagent (Bio-Rad Laboratories) with BSA as a standard. The purity of the nsP2-FL and nsP2-Pro proteins was evaluated by SDS/PAGE.

### Enzyme kinetic characterization

Three synthetic fluorescent substrates were purchased from Peptides International, Inc (Table 1). These substrates corresponded to nsP1/nsP2 (AGA), nsP2/nsP3 (AGC) and nsP3/nsP4 (AGG) cleavage sites of the chikungunya viral non-structural polyprotein. The amino acid substrates spanned P4-P'5 of each scissile site. The synthetic protein substrates were tagged with 2-(*N*-methylamino)benzoyl (Nma) groups at the N-terminus and 2,4-dinitrophenyl (Dnp) groups at an added lysine residue at the C-terminus. The AGA and AGG peptide substrates were dissolved with dimethyl sulfoxide (DMSO) whereas the AGC substrate was dissolved in dimethylformamide (DMF). Briefly, the stock substrate solutions were diluted in assay buffer (50 mM Tris/HCl, pH 7.5, 0.2 M arginine and 0.2 M glutamic acid) at varying concentrations. Then 90 μl of diluted substrates were pipetted into black 96-well plates. The reactions were started by adding 1 μM (10 μl) of the purified enzyme. The protease activity was continuously measured for substrate depletion using a Beckman Coulter DTX880 multimode detector at 340 nm excitation and 430 nm emission wavelengths at 37°C for 3 h. The progress curves (after subtraction of no enzyme controls) were analysed by Dynafit program^©^ version 3.28.070 (BioKin, Ltd.) to obtain the kinetic parameters [21].

**Table 1:** Cleavage site sequences of chikungunya virus nsP2 protease Three synthetic fluorescent substrates designated as AGA, AGC and AGG as underlined were synthesized corresponding to the cleavage site sequence of chikungunya virus nsP2 protease (nsP1/2, nsP2/3 and nsP3/4), respectively. 2-(*N*-methylamino)benzoyl (Nma) fluorophore group attached at the amino terminus and a 2,4-dinitrophenyl (Dnp) group attached to the carboxyl terminus of Lysine (K) residue.

Cleavage site (P4–P5′)	Recognition site	Designated name
nsP1/2	Nma-**RAGA**/**GIIET**k(Dnp)-OH	AGA
nsP2/3	Nma-**RAGC**/**APSYR**k(Dnp)-OH	AGC
nsP3/4	Nma-**RAGG**/**YIFSS**k(Dnp)-OH	AGG

### Protease activity characterization

The effects of salt, metal ions and protease inhibitors on nsP2 protease activity were biochemically characterized. Various concentrations of sodium chloride (NaCl) ranging from 100 mM to 2 M were used. The metal ions cobalt (Co^2+^), magnesium (Mg^2+^), zinc (Zn^2+^), nickel (Ni^2+^) and copper (Cu^2+^) were used at 2 mM in this study. Four different protease inhibitors were also tested at different final concentrations, 50 μM chymostatin (which inhibits chymotrypsin-like proteases), 10 μM E-64 (a highly selective cysteine protease inhibitor), 100 μM leupeptin (an inhibitor of serine and thiol proteases) and 1 mM phenylmethanesulfonyl fluoride (PMSF; a commonly used serine protease inhibitor). The enzyme reactions were performed as mentioned above with all three synthetic fluorescent substrates. The initial rate reaction was evaluated using GraphPad Prism® software, version 5.01. Percent remaining activity was calculated using the control activity in the absence of an inhibitor and the enzyme activity obtained in the presence of the inhibitor.

### Thermal stability test

The freshly purified enzymes were incubated at 37, 42, 50 and 60°C for 10 min and immediately measured for enzyme activity using the synthetic fluorescent AGG substrate. The initial reaction rate was evaluated using GraphPad Prism® software, version 5.01. Percent remaining activity was evaluated by comparing with the control reaction at 37°C.

## RESULTS

### nsP2 protein expression and purification

**Figure 1:**
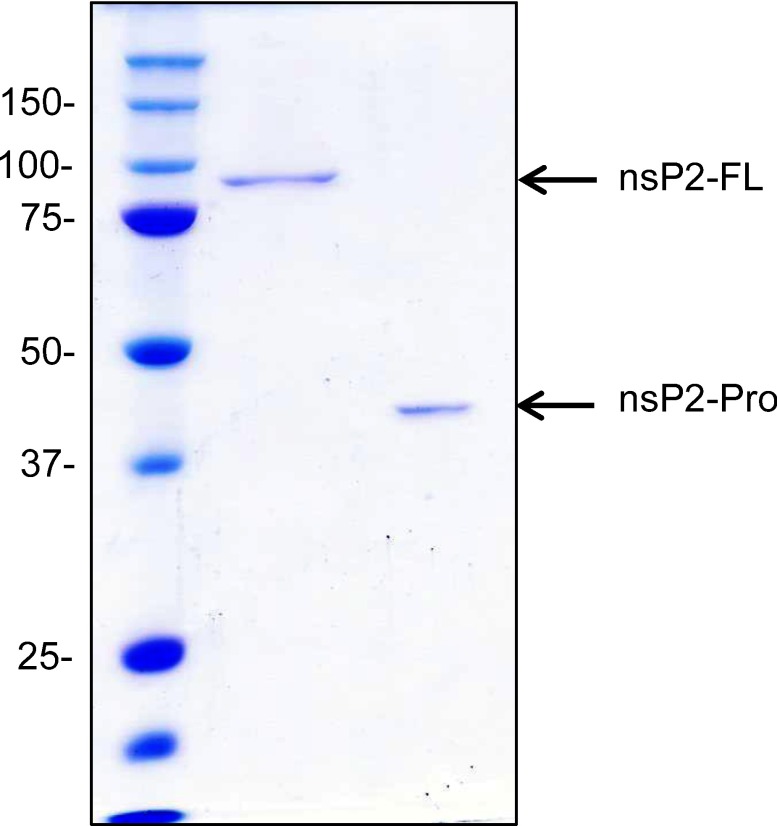
SDS/PAGE showing the purified recombinant CHIKV nsP2-FL and nsP2-Pro The two proteins (1 μg each) were loaded on SDS/PAGE (10%) and electrophoresis performed. The broad range markers sizes are shown on the left.

Three different expression vectors; His-tag, NusA-His-tag and GST-His-tag fusion, were initially tested as vectors for the expression of the nsP2-FL and nsP2-Pro proteins (data not shown). Results consistently showed that the nsP2 proteins were expressed as inclusion bodies under ‘high’ temperature culturing conditions (37°C), and only partially expressed in a soluble form at 22°C. Although lower temperatures and longer times of expression were investigated product solubility was only improved when a MBP-His fusion tag was added. The approximately 135 kDa (nsP2-FL) and 89 kDa (nsP2-Pro) fusion tag proteins were then expressed for subsequent purification. Several chromatography methods were employed in addition to MBPTrap and HisTrap columns. Moreover various metal ions including Ni^2+^, Co^2+^, Cu^2+^, Zn^2+^ and Ca^2+^ were also employed to charge the HisTrap columns. Unfortunately, both nsP2-fusion tag proteins tended to aggregate with contaminating *E. coli* proteins in all purifications steps (data not shown). Therefore additives to the purification buffers were investigated to dissociate co-eluting aggregate proteins. Finally, a mixture of arginine and glutamic acid that could significantly decrease the protein aggregation was found [22]. HisTrap columns charged with Ni^2+^ in the presence of 0.2 M arginine and 0.2 M glutamic acid gave the best partial purification from the first column. After cleaving the fusion enzyme with PreScission protease, the nsP2-FL and nsP2-Pro showed as approximately 90 and 43 kDa respectively on SDS/PAGE gels. The few contaminating proteins were subsequently removed by cation exchange column chromatography. It has to be stressed that the purification buffer of all steps contains equal molar concentrations of 0.2 M arginine and glutamic acid. The purified enzymes showed ~95% purity on SDS/PAGE gels stained with Coomassie Brilliant Blue R-250 (Figure 1).

### Kinetic parameter determination

Initially, trimeric chromogenic substrates linked to *p*-nitroaniline (*p*Na) were used in the protease assay, but protease activity could not be detected with these short chromogenic substrates (data not shown) and thus longer fluorescent peptide substrates were employed. The protease activity was then optimized and analysed according to Michaelis–Menten kinetics. We found that the fluorescent absorption became non-linear at high concentrations of substrates, a phenomenon known as the “inner filter effect” [23]. This problem was overcome by using lower substrate concentrations and analysing the data by progress curve analysis that conforms to the classical Michaelis–Menten equation. The kinetic parameters were then calculated by the Dynafit program [24,25].

Kinetic experiments were performed by varying concentrations of the three substrates. The turn-over number or *k*_cat_ of the two enzymes for each of the three substrates appear to be similar with both enzymes showing the lowest catalytic rate for the AGG (P3/4) substrate, which is the reported first cleavage site of the viral polyprotein (Table 2). The nsP2-FL *K*_m_ values are quite similar to one another, whereas the values vary up to 8-fold for the nsP2-Pro. This shows that the two enzymes have different binding affinity for the three substrates. These *K*_m_ values also are reflected in the catalytic efficiency (*k*_cat_/*K*_m_) of the two enzymes for all three substrates with the nsP2-FL being similar for the three cleavage sequences whereas the nsP2-Pro showed up to a 6-fold variation in catalytic efficiency.

**Table 2:** Kinetic parameters of nsP2-FL and nsP2-Pro The kinetic parameters were determined using progress curve analysis. The data were calculated by Dynafit program. The kinetic results are represented as mean ± S.D. for at least three independent experiments.

Substrates (cleavage site)	*k*_cat_ (10^−4^ S^−1^)	*K*_m_ (μM)	*k*_cat_/*K*_m_ (10^−4^ S^−1^ ·μM ^−1^)
nsP2-FL			
AGA (P1/2)	27.28±3.14	9.31±0.66	2.93
AGC (P2/3)	34.21±19.27	9.87±7.80	3.47
AGG (P3/4)	19.04±1.66	8.95±3.32	2.13
nsP2-Pro			
AGA (P1/2)	33.20±6.35	36.37±3.64	0.91
AGC (P2/3)	29.21±14.50	4.53±0.19	6.54
AGG (P3/4)	11.43±1.63	7.40±1.16	1.58

### The effect of salt, metal ion and protease inhibitors

**Figure 2:**
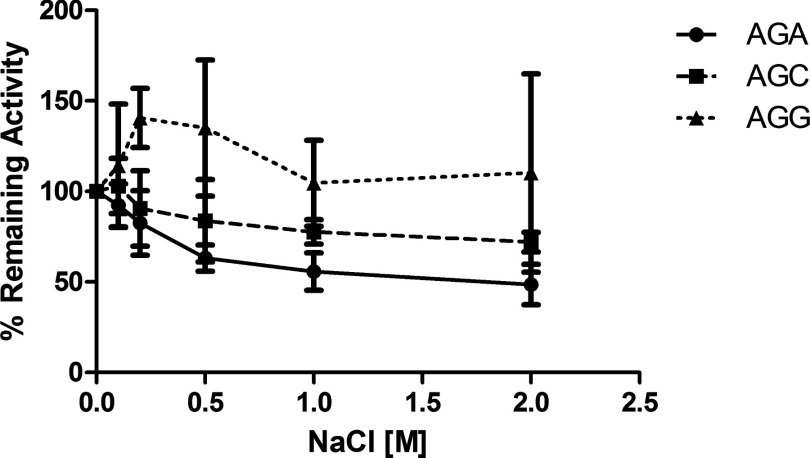
Salt effects on nsP2-Pro activity Sodium chloride concentrations ranging from 100 mM to 2 M were added to the nsP2-Pro protease activity assay.

**Figure 3:**
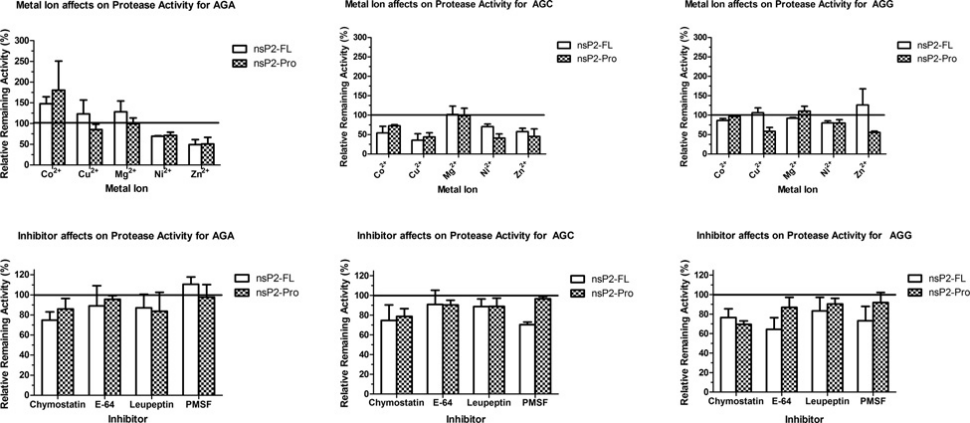
The effects of metal ions and protease inhibitors on nsP2 for full length and Pro activity The metal ions; cobalt (Co^2+^), magnesium (Mg^2+^), zinc (Zn^2+^), nickel (Ni^2+^) and copper (Cu^2+^) were used at 2 mM. Four different protease inhibitors were also tested at different final concentrations, 50 μM chymostatin (inhibits chymotrypsin-like proteases), 10 μM E-64 (highly selective cysteine protease inhibitor), 100 μM leupeptin (inhibitor of serine and thiol proteases) and 1 mM phenylmethanesulfonyl fluoride (PMSF; a common serine protease inhibitor). The enzyme reaction was performed as mentioned above with all three synthetic fluorescent substrates. Percent remaining activity was calculated using control activity in the absence of inhibitor and the enzyme activity obtained in the present of inhibitor. The data are represented as mean ± S.D. for at least three independent experiments.

Various concentrations of monovalent salt (NaCl) ranging from 100 mM to 2 M were added to the protease activity assay. The enzyme activity of nsP2-FL was not affected by salt up to 2 M (data not shown). However, the nsP2-Pro showed 30–40% inhibition at 1 M and above for AGA (P1/2) and AGC (P2/3) but a variable increase in AGG (P3/4) activity (Figure 2). A characterization study with metal ions was also performed (Figure 3). Both enzymes behaved similarly in the metal ion study. Magnesium did not inhibit either nsP2-FL or nsP2-Pro activity for all three substrates in contrast with nickel which inhibited protease activity some 20–30%. When using AGC as substrate, copper, cobalt and zinc showed inhibition of both enzymes’ activity ranging from 40 to 60%, but these metal ions showed little to no effect on the protease activity when using AGG as a substrate. A curious effect observed was an apparent activation by cobalt for AGA (P1/2) activity for both enzymes. Zinc metal ions inhibited nsP2-Pro for all substrates but inhibited nsP2-FL activity only with the AGA and AGC substrates (Figure 3).

The effect of different known protease inhibitors on activity was also studied. Four different protease inhibitors were selected. E-64 is a highly selective cysteine protease inhibitor. PMSF is a commonly used serine protease inhibitor whereas leupeptin is specific to serine and thiol proteases and chymostatin inhibits chymotrypsin-like proteases. PMSF did not affect either enzyme when using AGA (P1/2) as a substrate, but using AGC (P2/3) as a substrate PMSF showed no effect on nsP2-Pro while showing 30% inhibition of nsP2-FL. However, in general, these protease inhibitors seemed to have a similar overall effect on both enzymes with inhibition of 10–40% of activity at the concentrations tested (Figure 3).

### Thermal stability test

**Figure 4:**
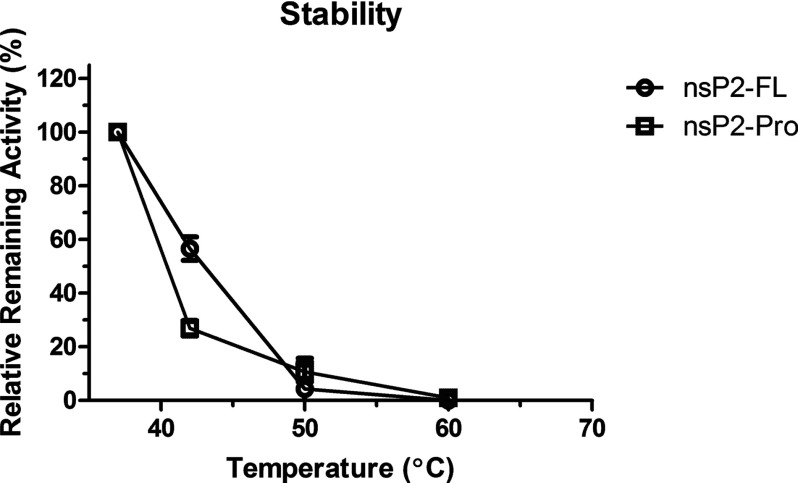
Thermal stability test The initial rate of the reactions was evaluated at different temperature; 37 (control), 42, 50 and 60°C. AGG substrate was used in this studied. The data represent the percent remaining activity of nsP2-FL compare to the activity at control temperature (37°C). The experiments were performed at least three independent experiments for each temperature.

The AGG fluorescent substrate was used in this experiment, and the control reaction was performed at 37°C. At 42°C, the activity of nsP2-FL decreased by 50% and nsP2-Pro lost 75% of activity. Both enzymes behaved similarly at 50°C and 60°C as shown in Figure 4.

## DISCUSSION

CHIKV nsP2 is an essential and multifunctional component of the viral replicase complex [10,26,27]. The protease role of nsP2 is critical for virus replication as only the virus protease activity is used for processing the viral non-structural polypeptide. To our knowledge, this is the first enzymatic characterization of the proteolytic activity of the full length nsP2 protein of CHIKV. Initially, most of the recombinant protein was expressed as insoluble inclusion bodies. The protein solubility problem also has been reported for expression of other alphavirus nsP2 proteins, for example the previously reported CHIKV nsP2-Pro, as well as from SFV and VEEV [11,28,29]. We overcame this by fusing the target proteins with an MBP fusion tag, an approach that has been reported to be useful for this problem [30]. After solving the solubility problem, we were able to purify the full length and Pro nsP2 proteins. Initially we found that the soluble proteins were very difficult to purify as the *E. coli* host proteins tended to aggregate with the recombinant nsP2. Interestingly, an equal molar mixture of arginine and glutamic acid helped reduce the interaction between proteins as well as improve the recombinant protein solubility. These two amino acids work synergistically by generating a ‘crowd’ of charged molecules on the protein surface preventing interactions with other proteins and increasing solubility [22].

Based on Schechter and Berger's protease nomenclature, the residues on the C-terminal of the scissile bond were assigned as prime site (P') whereas the residues on the N-terminal side were assigned as non-prime site (P) [31]. The residues at position P4–P1′ have been shown to be critical residues to enhance the protease activity and to have a high impact on substrate recognition for the alphavirus nsP2 from SFV [32]. This correlates with our early experiment in which we could not detect protease activity with short tri-peptide substrates. This poor proteoly-tic activity suggests that the flanking region of the scissile bond is very important in determining substrate specificity, and we therefore employed longer peptide substrates (Table 1). These three substrates represent the natural specific cleavage sites of the CHIKV nsP1234 polyprotein. Previous studies with an N-terminal truncated nsP2 (Pro39) protein of SFV showed that cleavage of substrates occurs in a specific manner and order. P3/4 (AGG) site is the first and the most efficient cleavage site as compared with P1/2 (AGA) and P2/3 (AGC) [33]. This implies that the nsP2 protease enzyme should have the highest *k*_cat_/*K*_m_ value toward AGG (P3/4) substrate, less for AGA (P1/2) and even lower activity for AGC (P2/3) substrate. Our characterization of CHIKV nsP2-FL and nsP2-Pro shows that these two enzymes are not only different from each other but are also different from the other reported alphavirus nsP2 proteases. The CHIKV nsP2-Pro showed a marked preference for AGC (P2/3) illustrated by its *k*_cat_/*K*_m_ value. In addition, the CHIKV nsP2 protease activity is in contrast with what has been reported previously for SIN, SFV and VEEV nsP2 proteases as no activity for these enzymes could be observed until cleavage substrates were fused to recombinant tag proteins like thioredoxin or maltose binding protein [33–35]. In fact, SFV nsP2-Pro and full length nsP2 generated no cleavage products if the substrate constructs had less than 10 amino acids on both sides of the sessile site [33]. It was also found that the nsP2-Pro showed greater activity than the nsP2 full length and there were large differences in specificity for the three cleavage sequences for both Pro and full length enzymes [33]. Therefore, it was suggested, the *in vivo* proteolytic processing is sequentially regulated based on the conformation of polypeptide or replication complex components [36,37]. The conformation of nsP1234 polyprotein may allow the P3/4 (AGG) cleavage site to be exposed on the surface of the polypeptide while the other sites are buried within the polypeptide chain. Once nsP2 protease cleaves the first P3/4 (AGG) cleavage site, this would affect the overall polyprotein conformation and increase the accessibility of the other cleavage sites. It has been suggested that the accessibility/cleavage would also be regulated by the replication complex, the complexes current constituents and the functional status of the complex [38]. Our data support this mechanism, as the kinetic values for each short cleavage sequence do not correlate with the order of the cleavage. We observed that the cleavage site requirements, especially for AGC (P2/3), differ for CHIKV nsP2 compared with the other reported alphavirus nsP2. It was reported that for cleavage to occur the N-terminus of nsP2 was required as well as the macro domain of nsP3, consisting of 170 N-terminal amino acids [38]. If these requirements are lacking then no cleavage of AGC (P2/3) occurs for nsP2 from SFV and SIN, whereas we show that both CHIKV nsP2-FL and nsP2-Pro have activity for a 9-mer AGC (P2/3) substrate.

**Figure 5:**
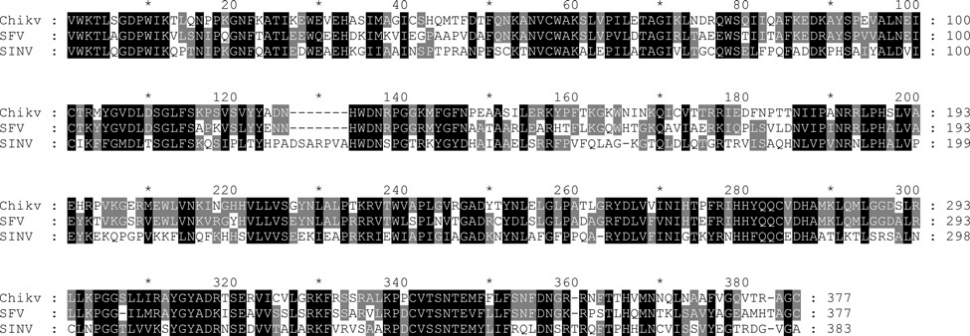
Amino acid alignment of the nsP2 protease domain from CHIKV, SFV and SINV The nsP2 amino acid sequences from CHIKV (ADK24721), SFV (NP_740666) and SINV (NP_740671) were aligned using the Genedoc program. The dashes indicate gaps in the sequence. Black shading indicates 100% conserved residues for the three sequences, dark grey indicates 80% conserved.

**Figure 6:**
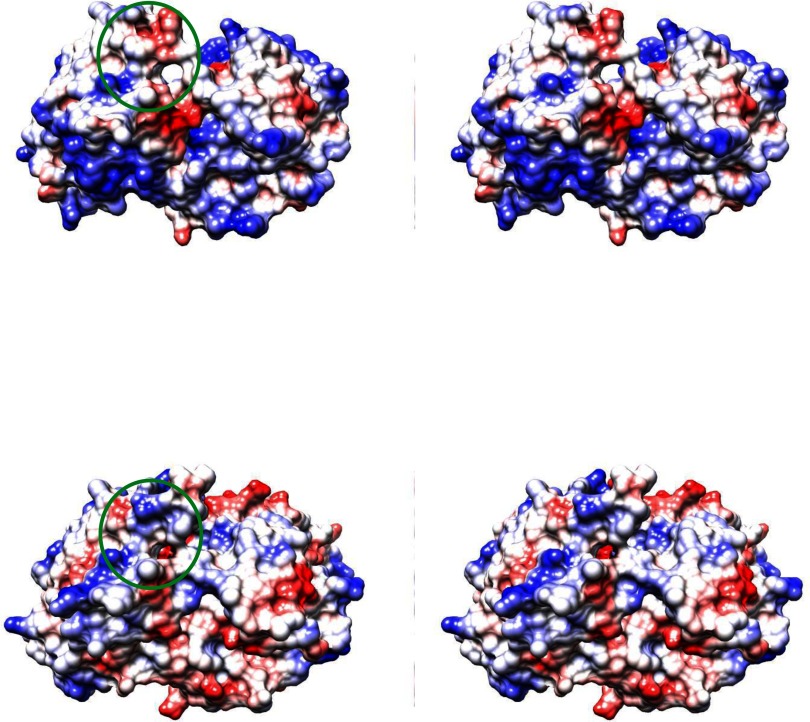
Stereo view of the electrostatic surface colouring of CHIKV nsP2-Pro and SINV nsP2-Pro The top panel is CHIKV nsP2-Pro (PDB ID: 3TRK) and the lower panel is SINV nsP2-Pro (PDB ID: 4GUA). The alpha carbons of the two structures were aligned with an RMSD of 1.024 Å (1 Å=0.1 nm) for 250 atom pairs. The active site catalytic dyad (Cys-His) is circled in green. Molecular graphics and analyses were performed with the UCSF Chimera package. Chimera is developed by the Resource for Biocomputing, Visualization, and Informatics at the University of California, San Francisco (supported by NIGMS P41-GM103311) [40].

An amino acid comparison of the full length nsP2 from the three alphaviruses, CHIKV, SFV and SINV, shows relatively high identity ranging from 57% to 71%. Upon closer examination it can be seen that the nsP2 N-terminus is more conserved than the C-terminal protease domain. CHIKV nsP2-Pro compared with the same region of SFV and SINV shows only 65% and 44% amino acid identity, respectively (Figure 5). Structural superposition of CHIKV nsP2-Pro (PDB ID: 3TRK) and SINV nsP2-Pro (PDB ID: 4GUA) illustrates the impact of this amino acid identity difference on the surface charge potential (Figure 6). Of particular interest is the difference shown in the substrate binding region of the two proteins where there are structural differences as well as charge potential differences. Obviously, these variances contribute to the substrate specificity differences observed.

We also characterized the impact of several metal ions and protease inhibitors on the behaviour of both full length and Pro nsP2. In general, both enzymes behaved similarly with two exceptions, Zn^2+^ appeared to activate the full length enzyme but inhibit the nsP2-Pro for AGG (P3/4) activity and PMSF appeared to inhibit full length but showed little effect on nsP2-Pro for AGC (P2/3).

To our knowledge, the previous alphavirus nsP2 characterization studies of inhibitor and metal ion effects reported have been performed with only a single substrate whereas we used all three substrates. This is of interest as the CHIKV nsP2 appears to behave differently with the test compounds depending on the substrate employed, for example, Co^2+^ increases AGA (P1/2) activity but inhibited AGC (P2/3) activity. Some of our results are in contrast with a previous report for the CHIKV nsP2-Pro [11]. However, Pastorino et al. [11] also showed kinetic properties varied for their enzyme with different peptide substrate lengths. We suggest that the peptide length, total as well as number of residues on each side of the scissile bond, and composition of the substrate which includes protein tags and chromogenic or fluorescent chromophores will impact upon the nsP2 catalytic specificity. Nevertheless, examples from the literature [35], and our present study supports the concept that the various alphavirus nsP2 proteases do display unique characteristics and the protease domain alone can exhibit different characteristics from the full length protein. This would appear to be of small consequence as the current understanding suggests that the virus polypeptide cleavage is regulated by the replication complex. However, the chikungunya nsP2 also appears to differ from other alphavirus nsP2 in its distinctive ability to recognize small peptide substrates. This is of greater potential interest as 50% of nsP2 is found in the nucleus after viral infection [39], and the functional significance of the localization is still unknown. Therefore an obvious issue to address in the future would be to determine whether there are host substrates for the CHIKV nsP2 protease.

## CONCLUSION

We show here for the first time the enzyme characterization of CHIKV nsP2 full length protein and compared it to the activity of the protease domain alone. The data show that the full length protein and the protease domain alone possess different kinetic properties and also that CHIKV nsP2 is different from other reported alphavirus nsP2 proteases in its ability to cleave small peptide substrates.

## References

[1] Ross R.W. (1956). The Newala epidemic. III. The virus: isolation, pathogenic properties and relationship to the epidemic. J. Hyg..

[2] Jupp P.G., Kemp A. (1996). What is the potential for future outbreaks of chikungunya, dengue and yellow fever in southern Africa?. S. Afr. Med. J./S.-Afr. Med. Tydskr..

[3] Singh S.K., Unni S.K. (2011). Chikungunya virus: host pathogen interaction. Rev. Med. Virol..

[4] Schwartz O., Albert M.L. (2010). Biology and pathogenesis of chikungunya virus. Nat. Rev. Microbiol..

[5] Vazeille M., Moutailler S., Coudrier D., Rousseaux C., Khun H., Huerre M., Thiria J., Dehecq J.S., Fontenille D., Schuffenecker I. (2007). Two Chikungunya isolates from the outbreak of La Reunion (Indian Ocean) exhibit different patterns of infection in the mosquito, Aedes albopictus. PloS One.

[6] Tsetsarkin K.A., Vanlandingham D.L., McGee C.E., Higgs S. (2007). A single mutation in chikungunya virus affects vector specificity and epidemic potential. PLoS Pathog..

[7] Johansson M.A. (2015). Chikungunya on the move. Trends Parasitol..

[8] Sourisseau M., Schilte C., Casartelli N., Trouillet C., Guivel-Benhassine F., Rudnicka D., Sol-Foulon N., Le Roux K., Prevost M.C., Fsihi H. (2007). Characterization of reemerging chikungunya virus. PLoS Pathog..

[9] Khan A.H., Morita K., Parquet Md Mdel C., Hasebe F., Mathenge E.G., Igarashi A. (2002). Complete nucleotide sequence of chikungunya virus and evidence for an internal polyadenylation site. J. Gen. Virol..

[10] Jose J., Snyder J.E., Kuhn R.J. (2009). A structural and functional perspective of alphavirus replication and assembly. Future Microbiol..

[11] Pastorino B.A., Peyrefitte C.N., Almeras L., Grandadam M., Rolland D., Tolou H.J., Bessaud M. (2008). Expression and biochemical characterization of nsP2 cysteine protease of Chikungunya virus. Virus Res..

[12] Karpe Y.A., Aher P.P., Lole K.S. (2011). NTPase and 5’-RNA triphosphatase activities of chikungunya virus nsP2 protein. PLoS One.

[13] Gomez de Cedron M., Ehsani N., Mikkola M.L., Garcia J.A., Kaariainen L. (1999). RNA helicase activity of Semliki Forest virus replicase protein NSP2. FEBS Lett..

[14] Hardy W.R., Strauss J.H. (1989). Processing the nonstructural polyproteins of sindbis virus: nonstructural proteinase is in the C-terminal half of nsP2 and functions both in cis and in trans. J. Virol..

[15] Merits A., Vasiljeva L., Ahola T., Kaariainen L., Auvinen P. (2001). Proteolytic processing of Semliki Forest virus-specific non-structural polyprotein by nsP2 protease. J. Gen. Virol..

[16] Russo A.T., White M.A., Watowich S.J. (2006). The crystal structure of the Venezuelan equine encephalitis alphavirus nsP2 protease. Structure.

[17] Russo A.T., Malmstrom R.D., White M.A., Watowich S.J. (2010). Structural basis for substrate specificity of alphavirus nsP2 proteases. J. Mol. Graphics Model..

[18] Strauss E.G., De Groot R.J., Levinson R., Strauss J.H. (1992). Identification of the active site residues in the nsP2 proteinase of Sindbis virus. Virology.

[19] ten Dam E., Flint M., Ryan M.D. (1999). Virus-encoded proteinases of the Togaviridae. J. Gen. Virol..

[20] Wikan N., Sakoonwatanyoo P., Ubol S., Yoksan S., Smith D.R. (2012). Chikungunya virus infection of cell lines: analysis of the East, Central and South African lineage. PloS One.

[21] Kuzmic P. (1996). Program DYNAFIT for the analysis of enzyme kinetic data: application to HIV proteinase. Anal. Biochem..

[22] Shukla D., Trout B.L. (2011). Understanding the synergistic effect of arginine and glutamic acid mixtures on protein solubility. J. Phys. Chem. B..

[23] Palmier M.O., Van Doren S.R. (2007). Rapid determination of enzyme kinetics from fluorescence: overcoming the inner filter effect. Anal. Biochem..

[24] Kuzmic P. (2009). Application of the Van Slyke-Cullen irreversible mechanism in the analysis of enzymatic progress curves. Anal. Biochem..

[25] Kuzmic P. (2009). DynaFit–a software package for enzymology. Methods Enzymol..

[26] Strauss J.H., Strauss E.G. (1994). The alphaviruses: gene expression, replication, and evolution. Microbiol. Rev..

[27] Solignat M., Gay B., Higgs S., Briant L., Devaux C. (2009). Replication cycle of chikungunya: a re-emerging arbovirus. Virology.

[28] Rikkonen M., Peranen J., Kaariainen L. (1994). ATPase and GTPase activities associated with Semliki Forest virus nonstructural protein nsP2. J. Virol..

[29] Russo A.T., Watowich S.J. (2006). Purification, crystallization and X-ray diffraction analysis of the C-terminal protease domain of Venezuelan equine encephalitis virus nsP2. Acta Crystallogr. Sect. F.

[30] Hammarstrom M., Hellgren N., van Den Berg S., Berglund H., Hard T. (2002). Rapid screening for improved solubility of small human proteins produced as fusion proteins in Escherichia coli. Protein Sci..

[31] Schechter I., Berger A. (1967). On the size of the active site in proteases. I. Papain. Biochem. Biophys. Res. Commun..

[32] Lulla A., Lulla V., Tints K., Ahola T., Merits A. (2006). Molecular determinants of substrate specificity for semliki forest virus nonstructural protease. J. Virol..

[33] Vasiljeva L., Valmu L., Kaariainen L., Merits A. (2001). Site-specific protease activity of the carboxyl-terminal domain of Semliki Forest virus replicase protein nsP2. J. Biol. Chem..

[34] Golubtsov A., Kaariainen L., Caldentey J. (2006). Characterization of the cysteine protease domain of Semliki Forest virus replicase protein nsP2 by *in vitro* mutagenesis. FEBS Lett..

[35] Zhang D., Tozser J., Waugh D.S. (2009). Molecular cloning, overproduction, purification and biochemical characterization of the p39 nsp2 protease domains encoded by three alphaviruses. Protein Expr. Purif..

[36] De Groot R.J., Hardy W.R., Shirako Y., Strauss J.H. (1990). Cleavage-site preferences of Sindbis virus polyproteins containing the non-structural proteinase. Evidence for temporal regulation of polyprotein processing *in vivo*. EMBO J..

[37] Shirako Y., Strauss J.H. (1990). Cleavage between nsP1 and nsP2 initiates the processing pathway of Sindbis virus nonstructural polyprotein P123. Virology.

[38] Lulla A., Lulla V., Merits A. (2012). Macromolecular assembly-driven processing of the 2/3 cleavage site in the alphavirus replicase polyprotein. J. Virol..

[39] Rikkonen M., Peranen J., Kaariainen L. (1992). Nuclear and nucleolar targeting signals of Semliki Forest virus nonstructural protein nsP2. Virology.

[40] Pettersen E.F., Goddard T.D., Huang C.C., Couch G.S., Greenblatt D.M., Meng E.C., Ferrin T.E. (2004). UCSF Chimera–a visualization system for exploratory research and analysis. J. Comput. Chem..

